# Frequency and predictors of anxiety and depression among pregnant women attending tertiary healthcare institutes of Quetta City, Pakistan

**DOI:** 10.1186/s12905-017-0411-1

**Published:** 2017-07-25

**Authors:** Rahila Ghaffar, Qaiser Iqbal, Adnan Khalid, Fahad Saleem, Mohamed Azmi Hassali, Nosheen Sikandar Baloch, Fiaz ud Din Ahmad, Sajid Bashir, Sajjad Haider, Mohammad Bashaar

**Affiliations:** 1 Faculty of Pharmacy & Health Sciences, University of Balochistan, Quetta, Pakistan; 2 Combined Military Hospital, Quetta, Pakistan; 3 School of Pharmaceutical Sciences, Universiti Sains Malaysia, Penang, Malaysia; 4 Gynecology & Obstetric Department, Bolan Medical College Hospital, Quetta, Pakistan; 5 Faculty of Pharmacy & Alternate Medicine, The Islamia University, Bahawalpur, Pakistan; 6 Faculty of Pharmacy, University of Sargodha, Punjab, Pakistan; 7 SMART Afghan International Trainings & Consultancy, Kabul, Afghanistan

**Keywords:** Anxiety and depression, Quetta city, Pakistan, Predictors, Pregnant women

## Abstract

**Background:**

Anxiety and depression (A&D) are commonly reported among pregnant women from all over the world; however, there is a paucity of workable data from the developing countries including Pakistan. The current study, therefore, aims to find out the frequency and predictors of A&D among pregnant women attending a tertiary healthcare institutes in the city of Quetta, in the Balochistan province, Pakistan.

**Methods:**

A questionnaire based, cross-sectional survey was conducted. The pre-validated Hospital Anxiety and Depression Scale (HADS) were used to assess the frequency of A&D among study respondents. Anxiety and depression scores were calculated via standard scoring procedures while logistic regression was used to identify the predictors of A&D. SPSS v. 20 was used for data analysis and *p* < 0.05 was taken as significant.

**Results:**

Seven hundred and fifty pregnant women responded to the survey. The majority of the respondents belonged to age group of 26–35 year (424, 56.4%) and had no formal education (283, 37.6%). Furthermore, 612 (81.4%) of the respondents were unemployed and had urban residencies (651, 86.6%). The mean anxiety score was 10.08 ± 2.52; the mean depression score was 9.51 ± 2.55 and the total HADS score was 19.23 ± 3.91 indicating moderate A&D among the current cohort. Logistic regression analysis reported significant goodness of fit (Chi square = 17.63, *p* = 0.030, DF = 3), indicating that the model was advisable. Among all variables, age had a significant association when compared with HADS scores [adjusted OR (odds ratios) = 1.23, 95% CI = 1.13–1.62, *p* < 0.001].

**Conclusion:**

Moderate A&D was reported among the study respondents. Furthermore, age was highlighted as a predictor of A&D. The evidence from this study provides a motion of support programs for anxious and depressed pregnant women. The benefits of implementing good mental health in antenatal care have long-lasting benefits for both mother and infant. Therefore, there is a need to incorporate A&D screening in the existing antenatal programs.

## Background

Pregnancy and the transition to motherhood involve major physical and psychological changes in the expecting mothers. These changes can become linked to the development of anxiety and depression during the pregnancy [1, 2]. Furthermore, during this critical period, women often report lack of self-confidence and are susceptible to the untoward aspects of this major event in their lives. [3]. Although, majority of women often see pregnancy and motherhood as a means and a social indicator of self-fulfillment, quite a few of them view pregnancy negatively, mainly because of apprehensions and fears related to childbirth, and an inadequate preparation for the upcoming mothering responsibilities. [4]. Such negative perceptions can, regardless of the socioeconomic conditions, result in the development of clinical anxiety and depression (A&D) before and up to a year after childbirth, [3].

Anxiety is characterized by an unpleasant state of inner turmoil, while depression is a state of low mood and aversion to physical activities that affects a person’s thoughts, behavior, feeling and sense of well-being [5, 6]. Ante partum A&D is a major public health problem globally and A&D disorders are highly prevalent during pregnancy [7–9]. In comparison to men, women in reproductive age group are reported to have double the frequency of depressive illnesses [10]. Women who experience a depressive illness during their first pregnancy are at greater risk for developing repeat episodes of depression, not only during their subsequent pregnancies but for the rest of their lives [11, 12]. For the past few decades, A&D during pregnancy has achieved greater attention as a possible risk factor for the development of postnatal complications with adverse infant outcomes and poor mother-baby bonding [13].

The prevalence of A&D during pregnancy varies from 10 to 20% in developed countries and is rated as the most established psychiatric disorders during pregnancy [14–17]. This empirical data authenticates that mood state, neuro-endocrine and immune system may play a critical role in the reproductive outcomes and fetal development [18]. Unfortunately, mental health is not acknowledged and has not received enough attention in developing countries [19]. The World Health Organization reported that almost one-in-three to one-in five pregnant women experiences a significant mental health problem in the developing countries [20]. Furthermore, it was also reported that such high rates of A&D were because of poor socio-economic development of the population, physical and psychological abuse and violence, and paucity of good health care delivery systems especially the mental health facilities [20].

Higher rates of A&D are reported from the developing countries. A prevalence of 29% (ante partum anxiety) and 18% (ante partum depression) was reported from a population-based study in rural Bangladesh [21]. Among the South Indian women, prevalence of depression during the last trimester was found to be 16% [22]. A study conducted in an urban area of Pakistan reported that 18% of the pregnant women were anxious and depressed during their pregnancies [23]. Another study conducted at the antenatal clinic of a teaching hospital in Lahore, Pakistan reported that 25% of the women suffered from depression and 34.5% from anxiety during their pregnancy [24]. It is obvious from these studies that pregnancy related A&D is highly prevalent in the developing countries.

A number of factors related to the development of pregnancy associated A&D have been reported in the literature [25–27]. Where Lancaster and colleagues reported stress, lack of social support and domestic violence as significant predictors of A&D during pregnancy [28], Kazi et al. reported increasing age and lower educational levels to be significantly associated with A&D with pregnancy [29].

To the best of our knowledge, A&D during pregnancy has not been reported from rural parts of Pakistan. Keeping in view that A&D rates are high in urban areas, we hypothesize that severity of A&D would be higher in the remote areas of Pakistan. Consequently, this study aims to explore the frequency of A&D among pregnant women attending public healthcare facilities in a low-income region, particularly Quetta, Pakistan. This explorative study also aims to identify the predictors of A&D among pregnant women.

## Methods

### Study design, settings and duration

A cross-sectional, observational study was planned for this research. Pregnant women attending Gynecology Out Patient Departments (OPDs) for their routine antenatal checkups at four different tertiary care hospitals namely Sandeman Provincial Hospital (SPH), Bolan Medical Complex Hospital (BMCH), Mohtarma Shaheed Benazir Bhutto Hospital (MSBBH) and Sheikh Khalifa Bin Zayed Hospital (SKZH) were targeted for data collection. These four hospitals are the biggest government hospitals of Quetta city and provide major healthcare facilities to the general population. The Obstetrics and Gynaecology (O&G) departments are well established in these hospitals, and all facilities and modern machinery are available. The total number of deliveries in the year 2015 at all four institutes were 23,236. Bolan Medical Complex Hospital shared the burden with 12,238 deliveries followed by SPH with 10,959 deliveries. Sheikh Khalifa Bin Zayed Hospital had 106 while MSBH had 43 deliveries. The study was conducted from August 2015 to July 2016.

### Study tool

The Hospital Anxiety Depression Scale (HADS) was used to determine A&D in the current cohort of the respondents. The HADS was originally developed by Zingmond and Snaith and is commonly used to determine the level of A&D among pregnant women. The HADS is a fourteen itemed scale that generates ordinal data whereby seven items relate to anxiety and seven items are related to depression. Each item scoring is 0–3 and the score for the entire scale ranges from (0–42) with high scores indicating higher A&D among the respondents [30]. The HADS has been thoroughly evaluated for cultural and standard validity in Pakistan [31]. Therefore, validated HADS in Urdu language (Language Franca) was used for interviewing our participants [32]. However, a pilot test was conducted before starting the official survey to ensure validity, logical sequence of questions, understanding and to establish a suitable time frame for the interview. The questionnaire was tested on 20 pregnant women and data were not included in the final analysis. The Cronbach alpha was used to assess the internal consistency of the research tool. The tool was declared reliable with alpha value of 0.80.

### Simple size and inclusion criteria

Pregnant women aged 15 years and above and having ability to communicate in Urdu (Language Franca) were targeted for the study. Patients having a previous history of A&D, on antidepressants for any other reasons, immigrants and not willing to participate were excluded from the study.

Determination of the sample size was carried out to ensure the minimum number of the respondents needed to be a representative sample of the whole population of Quetta city. By using a population based method through a double design effect, seven hundred and eighty four respondents were targeted for the study [33]. The figure (*n* = 784) was based on 95% confidence interval, 5% margin of error and 10% of dropout added to the final sample.

### Data collection and analysis

In addition to HADS, a structured questionnaire was used to get basic information about the respondents, including socio-demographic parameters and other relevant information. All respondents were interviewed in a private area. To avoid bias, family members of the respondents were not allowed to participate or intervene during the interview. The responses were coded and analyzed by using IBM Statistical Package Social Sciences (SPSS) v. 20.0. The Kolmogorov–Smirnov (KS) test was used for normality assessment and non-normal tests were used accordingly. Descriptive analysis was conducted whereby frequencies and percentages were used to describe demographic characteristics. The HADS was scored by using recommended methods of the developers. The Chi Square test was used to determine the association among study variables while logistic regression was used to identify predictor of A&D in our study respondents.

## Results

### Demographic characteristics of the study respondents

Table 1 shows the demographic characteristics of the study respondents. Seven hundred and fifty two women participated with a response rate of 95.1%. The majority of the respondents belonged to age group of 26–35 year (424, 56.4%) and had no formal education. Furthermore, the majority of participates were unemployed (612, 81.4%) and were living in an urban locality (651, 86.6%).

**Table 1:** Demographics characteristics of the study respondents

Demographics	Frequency	Percentage
Age		
16–25	177	23.5
26–35	424	56.4
36–45	151	20.1
Education		
No education	283	37.6
Primary	155	20.6
Secondary	195	25.9
Tertiary	119	15.8
Occupation		
Employed	132	17.6
Unemployed	612	81.4
Monthly income		
No income	6	0.8
> 5000	84	11.2
5000–15,000	321	42.7
< 15,000	341	45.3
Locality		
Urban	651	86.6
Rural	93	12.4
Ethnic group		
Urdu	110	14.6
Punjabi	121	16.1
Pathan	266	35.4
Baloch	173	23.0
Hazara	59	7.8
Others	23	3.1
Number of children		
0	35	4.7
1–5	589	78.3
6–10	120	15.9
> 10	8	1.1

Table 2 presents respondents’ response towards HADS. Four hundred and ninety (65.2%) respondents felt tensed from time to time. When participants were asked about being feeling cheerful during pregnancy, three hundred and ninety one (52.0%) responded as ‘not often’. Four hundred and twenty six (56.6%) respondents felt ‘not very much’ restless when they have to be on move while 252 (60.1%) respondents stated that they often felt sudden pain during pregnancy.

**Table 2:** Respondents to study questionnaire

Items	Frequency	Percentage
*I feel tense and ‘wound up’:*		
Most of the time	46	6.1
A lot of the time	207	27.5
From time to time, occasionally	490	65.2
Not at all	9	1.2
*I still enjoy the things I used to enjoy:*		
Definitely as much	129	17.2
Not quite as much	365	48.5
Only a little	226	30.1
Hardly at all	32	4.3
*I get a sort of frightened feeling as if something awful is about to happen:*		
Very definitely and quite badly	88	11.7
Yes, but not too badly	326	43.4
A little, but it doesn’t worry me	238	31.6
Not at all	100	13.3
*I can laugh and see the funny side of things:*		
As much as I always could	178	23.7
Not quite so much now	405	53.9
Definitely not so much now	149	19.8
Not at all	20	2.7
*Worrying thoughts go through my mind:*		
A great deal of the time	64	8.5
A lot of the time	218	29.0
From time to time, but not too often	374	49.7
Only occasionally	96	12.8
*I feel cheerful:*		
Not at all	18	2.4
Not often	391	52.0
Sometimes	299	39.8
Most of the time	44	5.9
*I can sit at ease and feel relaxed:*		
Definitely	150	19.9
Usually	369	49.1
Not often	213	28.3
Not at all	20	2.7
*I feel as if I am slowed down:*		
Nearly all the time	84	11.2
Very often	243	32.3
Sometimes	374	49.7
Not at all	51	6.8
*I get a sort of frightened feeling like “butterflies” in the stomach:*		
Not at all	106	14.1
Occasionally	345	45.9
Quite often	262	34.8
Very often	39	5.2
*I have lost interest in my appearance:*		
Definitely	77	10.2
I don’t take as much care as I should	255	33.9
I may not take quite as much care	280	37.2
I take just as much care as ever	140	18.6
*I feel restless as I have to be on the move:*		
Very much indeed	64	8.5
Quite a lot	175	23.3
Not very much	426	56.6
Not at all	87	11.6
*I look forward with enjoyment to things:*		
As much as I ever did	131	17.4
Rather less than I used to	453	60.2
Definitely less than I used to	140	18.6
Hardly at all	28	3.7
*I get sudden feeling of panic:*		
Very often indeed	107	14.2
Quite often	252	60.1
Not very often	147	19.5
Not at all	46	6.1
*I can enjoy a good book or radio or TV program:*		
Often	195	25.9
Sometimes	331	44.0
Not often	126	16.8
Very seldom	99	13.2

Mean anxiety score was 10.08 ± 2.52, mean depression score was 9.51 ± 2.55 and total HADS score was 19.23 ± 3.9 indicating moderate anxiety and depression among the current cohort

Tables 3 and 4 shows association among demographic and study questions. Respondents were asked about their feeling of getting tensed and wound up during pregnancy whereby age (*p* < 0.001) and number of children (*p* < 0.0011) were significantly associated with the statement. Furthermore, the Cramer’s V reported weak positive association between the two variables (φc = 0.192 and 0.180 respectively). Those participants who felt cheerful during pregnancy had age (*p* = 0.006), monthly income (*p* = 0.001), and number of children (*p* < 0.001) associated with the question and had moderate positive effect size (φc = 0.215, 0.225 and 0.213 respectively). When respondents were asked about their feeling of getting sudden panic during pregnancy, a weak positive association was reported as monthly income (*p* = 0.001) and age (*p* = 0.007) were significantly associated with the statement (φc = 0.198 and 0.167). Furthermore, those participants who were enjoying a good book or radio and T.V program during pregnancy reported to have significant association with age (*p* = 0.003, φc = 0.212), education (*p* < 0.001, φc = 0.243) and monthly income (*p* = 0.004, φc = 0.245). Although there were some other significant associations (Table 3), the effect size was too little to produce a significant outcome of the model. For other variables, no association was reported among other variables and study questions.

**Table 3:** Association between study questions and demographic variables

Question	*P*-value**						
	Age	Education	Occupation	Monthly Income	Locality	Ethnicity	No of children
I feel tense and ‘wound up’:	0.021* φc = 0.192	0.710	0.494	0.245	0.418	0.604	< 0.001* **φc = 0.180**
I still enjoy the things I used to enjoy:	0.003* **φc = 0.012**	0.064	0.523	0.918	0.457	0.719	0.006* **φc = 0.092**
I get a sort of frightened feeling as if something awful is about to happen:	0.193	0.077	0.513	0.031	0.257	0.328	0.656
I can laugh and see the funny side of things:	0.432	0.087	0.339	0.913	0.322	0.601	0.305
Worrying thoughts go through my mind:	0.431	0.016* **φc = 0.100**	0.009* **φc = 0.088**	0.001* **φc = 0.101**	0.004* **φc = 0.133**	0.068	0.425
I feel cheerful:	0.006* φc = 0.215	0.913	0.282	< 0.001* φc = 0.225	0.101	0.856	< 0.001* **φc = 0.213**
I can sit at ease and feel relaxed:	0.676	0.173	0.343	0.335	0.656	0.309	0.630
I feel as if I am slowed down:	0.917	0.112	0.319	0.143	0.302	0.171	0.809
I get a sort of frightened feeling like “butterflies” in the stomach:	0.684	0.067	0.238	0.250	0.744	0.230	0.064
I have lost interest in my appearance:	0.075	0.131	0.254	0.724	0.194	0.204	0.117
I feel restless as I have to be on the move:	0.109	0.303	0.228	0.801	0.322	0.068	0.213
I look forward with enjoyment to things:	0.453	0.323	0.998	0.103	0.071	0.415	0.701
I get sudden feeling of panic:	0.007* **φc = 0.198**	0.264	0.341	0.001* **φc = 0.167**	0.443	0.072	0.200
I can enjoy a good book or radio or TV program:	0.003* **φc = 0.212**	< 0.001* **φc = 0.243**	0.667	0.004* **φc = 0.245**	0.525	0.228	0.355

*Phi/Cremer V (where appropriate; interpreted by Cohen, Jacob, 1988, Statistical power and analysis for the behavioral sciences (2nd ed.), Hillsdale, N.J., Lawrence Erlbaum Associates, Inc)

**Chi square test

**Table 4:** Logistic regression analysis*

Variables	Anxiety & Depression (A&D) %		OR (95% Cl)	*P*-value
	With A&D	Without A&D		
Age				
16–25	19.7	80.3	Ref	
26–35	7.5	92.5	1.01 (0.81–1.50)	0.231
36–45	41.0	59.0	1.23 (1.13–1.62)	*<0.001*
Education				
No education	16.2	83.8	Ref	
Primary	29.1	70.9	1.40 (0.70–2.12)	0.352
Secondary	18.9	81.1	1.03 (0.55–1.14)	0.299
Tertiary	22.6	77.4	1.19 (0.62–1.03	0.676
Occupation				
Employed	21.9	78.1	Ref	
Unemployed	19.2	80.8	0.90 (0.65–1.00)	0.893
Monthly income				
No income	16.6	83.4	Ref	
> 5000	23.8	76.2	0.33 (0.20–0.94)	0.089
5000–15,000	35.8	64.2	0.56 (0.34–0.88)	0.551
< 15,000	15.5	84.5	0.64 (0.17–0.99)	0.300
Locality				
Urban	17.9	82.1	Ref	
Rural	24.7	75.3	2.13 (1.96–3.11)	0.337
Ethnic group				
Urdu	10.9	89.1	Ref	
Punjabi	19.0	81.0	0.53 (0.23–0.98)	0.445
Pathan	19.1	80.9	0.49 (0.34–1.32)	0.690
Baloch	22.5	77.5	0.56 (0.45–1.00)	0.711
Hazara	16.9	83.1	0.44 (0.45–1.27)	0.49
Others	21.0	79.0	0.43 (0.30–0.88)	0.380
Number of children				
0	28.5	71.5	Ref	
1–5	28.9	71.1	1.56 (1.09–2.62)	0.997
6–10	44.1	55.9	1.78 (1.22–2.19)	0.560
> 10	75.0	25.0	1.98 (1.77–2.92)	0.558

*Significant goodness of fit (Chi square = 17.63, *p* = 0.030, DF = 3)

Logistic regression was used to identify the predictors of A&D. The HADS scores were dichotomized into ‘with stress/anxiety’ and ‘without stress/anxiety’. The model was reported as acceptable with high significant values (Chi square = 17.63, *p* = 0.030, DF = 3) with capability of 23.0–32.2% of the total variance explained. Among all variables, age shaped as a predicting variable against A&D (adjusted OR = 1.23, 95% CI = 1.13–1.62, *p* < 0.001). The model explained that an increase of 1% in age was associated with an increase of 1.23 in A&D provided all other variables remain constant.

## Discussion

The objective of the current study was to highlight the frequency and predictors of A&D among the current study respondents. In our study, 752 pregnant women participated with a total HADS score of 19.23 indicating A&D being moderately prevalent during pregnancy in Quetta city, Pakistan.

### Frequency of A&D

The rates of A&D during pregnancy vary around the globe, but as expected, it is higher in the developing nations [34]. A higher rate of A&D among pregnant women in Pakistan is reported in the literature [35]. A study conducted in antenatal clinic of a teaching hospital in Lahore, Pakistan reported that 25% of women suffered from depression and 34.5% from anxiety during pregnancy [24]. Rozina and colleagues in their study reported from Hyderabad, Pakistan concluded that 18% of the study participants were anxious and depressed during pregnancy [36]. In comparison, studies from the developed countries reported lower prevalence of A&D during pregnancy. For example, a nationwide survey conducted in the USA from 1996 to 2006 reported that prevalence of antenatal anxiety and depression in ten years was 7.8% [37]. Gaynes and colleagues in their systematic review of 109 articles reported 13% of pregnant women to suffer from major/minor depression [38], while a higher prevalence of depression (20%, according to the Beck Depression Inventory) and anxiety (60%, according to the State-Trait Anxiety Inventory) among pregnant women in Sao Paulo, Brazil in 2007 was also reported in the literature [39]. Furthermore, in the comparison of antenatal depression among Pakistani and Canadian women, Mahboob et al. found a higher prevalence of antenatal depression among Pakistani women (48%) when compared with 31% of Canadian aboriginal [40].

### Predictors of A&D

The other part of the study examined the different responses to the questionnaire based on seven socio-demographic variables. Though the responses were different from women belonging to different socioeconomic groups having varying levels of education and different number of children, age of the pregnant women was shown to be the most significant predictor for the development of A&D. This was also shown in another study conducted at Karachi, Pakistan, where increasing age was associated with increasing A&D in women [29] of the reproductive age group, pregnant and otherwise [41]. Our findings are also supported by another study conducted in China. The authors declared 30 years of age as a maximum to explore, because an increase in age influenced the level of A&D in pregnant women. Pregnancy in women aged more than 35 is perceived as highly risked that can lead to complications [42]. On the contrary, high rates of A&D among pregnant women aged less than 30 years is also reported in the literature [43] which is also supported by the findings of Pigott and colleagues [44]. Age is an important variable and an antenatal educational campaign must consider this variable before designing a learning program for the pregnant women.

Our study reported that unemployed women were more depressed during pregnancy. Unemployed women lack economic support and have a lot of free time to think about their pregnancy. Social support mechanisms serve as assistance in improving adaptation and emotional stability. It is reported that as social support increases psychological stress decreases [45]. Hence, we can conclude that pregnant women having low levels of social support experience higher rates of A&D. This conclusion is supported by our study results as employed women were less depressed than unemployed women during their pregnancy. Employment yet again was mentioned as a strong protective factor against major depression in pregnancy [46], which however is not supported by the current study results.

Studies from the developed world report higher frequency of psychiatric disorders among urban populations when compared with rural [47]. Findings from the current study reveal that 86.6% urban pregnant women were suffering from A&D. This may be explained by the unique environmental factors that pregnant women are exposed to in urban areas of developing countries. Firstly, there is a wide variation in the standards of living among the urban communities in developing countries. Secondly, even in some urban areas of Pakistan, there is lack of several basic facilities, including good healthcare services, clean drinking water, sanitation, and uninterrupted heating and power supply. The women in the rural areas on the other hand are less involved in important decision making and hence supposedly less exposed to the day to day stresses of life. It is hypothesized that rural pregnant women generally suffer less from A&D.

In our study those women were more depressed and anxious; whose number of children was 1–5. They may be depressed due to unplanned pregnancy or poverty may be a common source of A&D for pregnant women. One study conducted in Peshawar, Pakistan also reported a direct association between poverty and depression [48].

## Conclusion

The HADS score indicates moderate anxiety and depression among the current cohort from the region of Quetta, Pakistan. The evidence from this study provides a motion for starting effective social support programs for anxious and depressed pregnant women. A comprehensive health promotion program regarding maternal mental health should be introduced, on one hand to the pregnant women in antenatal clinics and on the other, to the public in the community. Legislatures and policy makers must setup strategies in place which should effectively transfer the required knowledge and awareness to the pregnant women, their husbands, families and friends so that they can understand the early warning signs and symptoms of A&D and thus prevent its adverse outcomes for the betterment of the whole community.

### Limitation

Our study is limited to four tertiary care settings and the results cannot be generalized. A nationwide study is required to get a clear picture of A&D among pregnant women in Pakistan.

## Abbreviations

A&D: Anxiety and depression
BMCH: Bolan Medical Complex Hospital
HADS: Hospital anxiety and depression scale
KS: Kolmogorov–Smirnov
MSBBH: Mohtarma Shaheed Benazir Bhutto Hospital
O&G: Obstetrics and Gynaecology
OPDs: Out Patient Departments
OR: Odds ratios
SKZH: Sheikh Khalifa Bin Zayed Hospital
SPH: Sandeman Provincial Hospital
SPSS: Statistical Package Social Sciences

## References

[1] Teixeira CF, Figueiredo B, Conde A, Pacheco A, Costa R (2009). Anxiety and depression during pregnancy in women and men. J Affect Disord.

[2] César T, Bárbara F, Ana C, Alexandra P, Raquel C (2009). Anxiety and depression during pregnancy in women and men. J Affect Disord.

[3] Bhat NA, Hassan R, Shafiq M, Sheikh S (2015). Sociodemographic factors: a major predictor of anxiety and depression among pregnant women. Delhi Psychiatry J.

[4] Fareeha H, Aftab A, Ijaz MI (2008). Study of anxiety and depression during pregnancy. Pak J Med Sci.

[5] Seligman MEP, Rosenhan DL (1989). Abnormal psychology.

[6] Sandra S (1997). Depression: question you have-answers you need.

[7] A global burden of disease: a comprehensive assessment of mortality and disability from diseases, injuries and risk factors in 1990 and projected to 2020. Available from: http://apps.who.int/iris/bitstream/10665/41864/1/0965546608_eng.pdf. Accessed 2 Jan 2017.

[8] Lovisi GM, López JR, Coutinho ES, Patel V (2005). Poverty, violence and depression during pregnancy: a survey of mothers attending a public hospital in brazil. Psychol Med.

[9] Ajinkya S, Jadhav PR, Srivastava NN. Depression during pregnancy: Prevalence and obstetric risk factors among pregnant women attending a tertiary care hospital in Navi Mumbai. Ind Psychiatry J. 2013;22(1):37. 10.4103/0972-6748.123615 PMC3895310 24459372

[10] Mental health aspects of women’s reproductive health: A global review of the literature. Available from: http://apps.who.int/iris/bitstream/10665/43846/1/9789241563567_eng.pdf. Accessed 3 Jan 2017.

[11] Surgeon general’s workshop on women’s mental health. Available from: https://www.ncbi.nlm.nih.gov/books/NBK44650/. Accessed 3 Jan 2017.

[12] Nonacs R, Cohen LS (2002). Depression during pregnancy: diagnosis and treatment options. J Clin Psychiatry.

[13] Burt VK, Stein K (2002). Epidemiology of depression throughout the female life cycle. J Clin Psychiatry.

[14] Costa DD, Larouche J, Dritsa M, Brender W (2000). Psychosocial correlates of prepartum and postpartum depressed mood. J Affect Disord.

[15] Evans J, Heron J, Francomb H, Oke S, Golding J (2001). Cohort study of depressed mood during pregnancy and after childbirth. Brit Med J.

[16] Bennett HA, Einarson A, Taddio A, Koren G, Einarson TR (2004). Prevalence of depression during pregnancy: systematic review. Obstet Gynecol.

[17] Strat YL, Dubertret C, Foll BL (2011). Prevalence and correlates of major depressive episode in pregnant and postpartum women in the United States. J Affect Disord.

[18] Kim HG, Mandell M, Crandall C, Kuskowski MA, Dieperink B, Buchberger RL (2006). Antenatal psychiatric illness and adequacy of prenatal care in an ethnically diverse inner-city obstetric population. Arch Womens Ment Health.

[19] Verbeek T, Arjadi R, Vendrik J, Burger H, Berger MY (2015). Anxiety and depression during pregnancy in central America: a cross-sectional study among pregnant women in the developing country Nicaragua. BMC Psychiatry.

[20] Maternal mental health and child health and development in low and middle income countries. Available from: http://www.who.int/mental_health/prevention/suicide/mmh_jan08_meeting_report.pdf. Accessed 29 Dec 2016.

[21] Nasreen HE, Kabir ZN, Forsell Y, Edhborg M (2011). Prevalence and associated factors of depressive and anxiety symptoms during pregnancy. BMC Womens Health.

[22] Chandran M, Tharyan P, Muliyil J, Abraham S (2002). Post- partum depression in a cohort of women from a rural area of Tamil Nadu, India.Incidence and risk factors. Br J Psychiatry.

[23] Karmaliani RAN, Bann CM, Moss N, Mcclure E, Pasha O, Wright LL, Goldenberg RL (2009). Prevalence of anxiety, depression and associated factors among pregnant women of Hyderabad. Int J Soc Psychiatry.

[24] Niaz S, Izhar N, Bhatti MR (2004). Anxiety and depression in pregnant women presenting in the opd of a teaching hospital. Pak J Med Sci.

[25] Faisal-Cury A, Menezes PR (2007). Prevalence of anxiety and depression during pregnancy in a private setting sample. Arch Womens Ment Health.

[26] Chen H, Chan YH, Tan KH, Lee T (2004). Depressive symptomatology in pregnancy. Soc Psychiatry Psychiatr Epidemiol.

[27] Christine R, Wickberg B, Gustavsson P, Ingela R (2005). Depressive symptoms in early pregnancy, two months and one year postpartum-prevalence and psychosocial risk factors in a national Swedish sample. Arch Womens Ment Health.

[28] Lancaster CA, Gold KJ, Flynn HA, Yoo H, Marcus SM, Davis MM (2010). Risk factors for depressive symptoms during pregnancy: a systematic review. Am J Obstet Gynecol.

[29] Kazi A, Fatmi Z, Hatcher J, Kadir MM, Niaz U, Wasserman GA (2006). Social environment and depression among pregnant women in urban areas of Pakistan: importance of social relations. Soc Sci Med.

[30] Zigmond AS, Snaith RP (1983). The hospital anxiety and depression scale. Acta Psychiat Scand.

[31] Ahmer S, Faruqui AR, Aijaz A (2007). Psychiatric rating scales in Urdu: a systematic review. BMC Psychiatry.

[32] Mumford DB, Tareen AK, Bajwa MAZ, Bhatti MR, Karim R (1991). The translation and evaluation of an Urdu version of the hospital anxiety and depression scale. Acta Psychiat Scand.

[33] Gorstein J, Sullivan KM, Parvanta I, Begin F: Indicators and methods for cross-sectional surveys of vitamin and mineral status of populations. The Micronutrient Initiative (Ottawa) and the Centers for Disease Control and Prevention (Atlanta) 2007. Available from: http://www.who.int/vmnis/toolkit/mcn-micronutrient-surveys.pdf. Accessed 2 Jan 2017.

[34] O'Keane V, Marsh SM (2007). Depression during pregnancy. Brit Med J.

[35] Hussain N, Creed F, Tomenson B (2000). Depression and social stress in Pakistan. Psychol Med.

[36] Karmaliani R, Asad N, Bann CM, Moss N, McClure ME, Pasha O, Wright LL, Goldenberg LR (2009). Prevalence of anxiety, depression and associated factors among pregnant women of Hayderabad, Pakistan. Int J Soc Psychiatry.

[37] Witt WP, Street NW, Hagen EW, Wichmann MA (2011). The prevalence and determinants of antepartum mental health problems among women in the USA: a nationally representative population-based study. Arch Womens Ment Health.

[38] Gaynes BN, Gavin N, Meltzer-Brody S, Lohr KN, Swinson T, Gartlehner G, Brody S, Miller WC (2005). Perinatal depression: prevalence, screening accuracy, and screening outcomes. AHRQ Evid Rep Summ.

[39] Faisal-Cury A, Rossi Menezes P (2007). Prevalence of anxiety and depression during pregnancy in a private setting sample. Arch Womens Ment Health.

[40] Mahboob S, Shah A, Bowen A, Afridi I, Nowshad G, Muhajarine N (2011). Prevalence of antenatal depression: comparison between Pakistani and Canadian women. J Pak Med Assoc.

[41] Ali BS, Rahbar MH, Naeem S, Tareen AL, Gul A, Samad L (2002). Prevalence of and factors associated with anxiety and depression among women in lower moddle class semi-urban community of Karachi, Pakistan. J Pak Med Assoc.

[42] Yu-ting K, Yan Y, Jing D, Xin G, Shu-Yue L, Cai-ning Z, Hong-zhi H, Bo L (2016). Prevanlence and risk factors of maternal anxiety in late pregnancy in China. Int J Environ Res Public Health.

[43] Bhat NA, Hassan R, Shafiq M, Sheikh S (2015). Sociodemographic facts: a major predictor of anxiety and depression among pregnant women. Delhi Psychiatry J.

[44] Pigott TA (2003). Anxiety disorders in women. Psychiatr Clin North Am.

[45] Ozbay F, Fitterling H, Charney D, Southwick S (2008). Social support and resilience to stress across the life span: a neurobiologic framework. Curr Psychiatry Rep.

[46] Fall A, Goulet L, Vézina M (2013). Comparative study of major depressive symptoms among pregnant women by employment status. Spring.

[47] Witt WP, Street NW, Hagen EW, Wichmann MA (2011). The prevalence and determinants of antepartum mental health problems among women in the USA: a nationally representative population-based study. Arch Womens Ment Heal.

[48] Patel V, Rodrigues M, DeSouza N (2002). Gender, poverty, and postnatal depression: a study of mothers in Goa, India. Am J Psychiatry.

